# Gene therapy for malignant glioma

**DOI:** 10.1186/2052-8426-2-21

**Published:** 2014-07-08

**Authors:** Hidehiro Okura, Christian A Smith, James T Rutka

**Affiliations:** The Arthur and Sonia Labatt Brain Tumour Research Centre, Peter Gilgan Centre for Research and Learning, The Hospital for Sick Children, 686 Bay Street, 17th Floor, Toronto, ON M5G 0A4 Canada; Department of Neurosurgery, Juntendo University School of Medicine, 2-1-1 Hongo, Bunkyo-ku, Tokyo, 113-8421 Japan; Department of Surgery, University of Toronto, 149 College Street, 5th Floor, Toronto, Ontario M5T 1P5 Canada; Division of Neurosurgery, The Hospital for Sick Children, Suite 1503, 555 University Avenue, Toronto, Ontario M5G 1X8 Canada

**Keywords:** Glioblastoma, Gene therapy, Prodrug suicide, Oncolytic, Cytokine mediated, Tumor suppressor gene

## Abstract

Glioblastoma multiforme (GBM) is the most frequent and devastating primary brain tumor in adults. Despite current treatment modalities, such as surgical resection followed by chemotherapy and radiotherapy, only modest improvements in median survival have been achieved. Frequent recurrence and invasiveness of GBM are likely due to the resistance of glioma stem cells to conventional treatments; therefore, novel alternative treatment strategies are desperately needed. Recent advancements in molecular biology and gene technology have provided attractive novel treatment possibilities for patients with GBM. Gene therapy is defined as a technology that aims to modify the genetic complement of cells to obtain therapeutic benefit. To date, gene therapy for the treatment of GBM has demonstrated anti-tumor efficacy in pre-clinical studies and promising safety profiles in clinical studies. However, while this approach is obviously promising, concerns still exist regarding issues associated with transduction efficiency, viral delivery, the pathologic response of the brain, and treatment efficacy. Tumor development and progression involve alterations in a wide spectrum of genes, therefore a variety of gene therapy approaches for GBM have been proposed. Improved viral vectors are being evaluated, and the potential use of gene therapy alone or in synergy with other treatments against GBM are being studied. In this review, we will discuss the most commonly studied gene therapy approaches for the treatment of GBM in preclinical and clinical studies including: prodrug/suicide gene therapy; oncolytic gene therapy; cytokine mediated gene therapy; and tumor suppressor gene therapy. In addition, we review the principles and mechanisms of current gene therapy strategies as well as advantages and disadvantages of each.

## Review

### Introduction

Gliomas are the most frequently occurring primary brain tumor in adults. Glioblastoma multiforme (GBM) is the most aggressive form and least curable [1]. The current standard of treatment consists of maximal surgical resection followed by radiation and temozolomide (TMZ) chemotherapy [2]. Despite recent reports that demonstrate a two-month survival advantage when adjuvant TMZ chemotherapy is used, the median survival still remains less than 15 months and death ensues in most cases within 2 years of diagnosis [3, 4]. The high mortality observed with GBM is due to a consequence of many contributing factors including the aggressive and invasive phenotype making radical surgical removal extremely difficult. In addition, GBM is often resistant to radiation and/or chemotherapy. Chemotherapy may fail because of an inability to effectively deliver reagents across the blood brain barrier (BBB). The central nervous system is also largely regarded as an immunologically privileged site, and protected from systemic immune responses. This presents a disadvantage with respect to the efficacy of systemic immune-boosting strategies.

It is now generally accepted that all cancers contain a small population of cells with stem cell like properties called cancer stem cells (CSCs). The concept of the CSC has been extended to brain tumors, including GBM, and it is now being exploited as a therapeutic target. Glioma stem cell (GSCs) are capable of asymmetric cell division into self-renewing GSCs and differentiating daughter cells that can gain different phenotypes, subsequently losing their multipotent property [5]. Disease progression and an inevitable recurrence after therapy are most likely attributed to GSCs which are highly invasive and resistant to radiotherapy and chemotherapeutic agents [6–8]. Unfortunately, despite the significant progress achieved by surgery and adjuvant chemoradiotherapy including molecular-targeted approaches in the treatment of disseminated malignancies, the prognosis of GBM remains unsatisfactory. Therefore, novel and more efficient strategies are urgently needed, and real progress can only emerge from increasing our understanding of the molecular biology of these tumours, and through the discovery of novel mechanisms for the delivery of tumoricidal agents.

Gene therapy can be defined as the treatment of disease by the introduction of a therapeutic gene or the manipulation of a disease-related gene such as abrogation of an activated oncogene within target cells [9]. Owing to a better understanding of the mechanisms of virus interactions with the cell and the advancement of recombinant deoxyribonucleic acid (DNA) technology, it is now possible to take advantage of the tumor cell-specific genetic defects and to construct viral strains that replicate selectively in tumor cells. To date, gene therapy has been applied to several types of cancer [10]. One of the most dismal types of cancer, GBM is an ideal target for gene therapy given that current standard therapies remain minimally effective and that GBM rarely metastasizes to other locations in the body.

The first clinical trials of gene therapy targeting gliomas were published in the 1990’s [11, 12]. Different methods, including: viral vectors; cellular carriers (neural stem cell, mesenchymal stem cell, or embryonic stem cell); and synthetic vectors using nanotechnology (nanoparticle or cationic liposome) have been studied and employed as a vehicle to deliver genes into the target cells. Although stem cells as vehicles have only recently reached clinical study, they promise to be one of the most attractive vectors to combine gene therapy with other type of therapies. The only synthetic vector that has reached clinical trials against glioma to date is a cationic liposome which was employed as a small molecule carrier [13, 14]. Although, gene transfer using liposomes is considered safe, it has been used infrequently due to limited gene transfer efficiency. On the other hand, viral vectors are considered to be the most effective of all gene delivery methods for *in vivo* gene transfer [15]. Currently, the most frequently used DNA delivery vehicles are genetically modified viruses or vectors. There are two types of viral vectors used for anti-glioma therapy. The first uses replication-deficient viruses capable of transducing genes into the tumor cells resulting in detrimental intracellular effects; and the second employs oncolytic viruses where the replicating viruses have a lytic cycle, and selectively kills tumor cells. Among all viral vectors, adenovirus (AV), retrovirus, herpes simplex virus (HSV), and adeno-associated virus (AAV) are currently the most widely employed delivery vectors used in gene therapy in patients with cancer [15, 16].

There are a wide variety of strategies for gene therapy of GBM. In addition to the large number of vectors and their individual features, different transgenes provide distinct ways of eliciting an anti-tumoral response. Consequently, gene therapy is a viable option for the treatment of GBM. In this article, we will review (i) the major approaches used for gene therapy against GBM including prodrug/suicide gene therapy, oncolytic gene therapy, cytokine mediated gene therapy, and tumor suppressor gene therapy; (ii) the rationale for the design of vectors; (iii) mechanisms of the vectors replication in tumor cells; (iv) discuss advantages and disadvantages of each gene therapies and future direction.

### Approaches to gene therapy

#### Suicide gene therapy

The most commonly used gene therapy strategy against malignant glioma in preclinical study and in clinical trials is suicide gene therapy [17]. Suicide gene therapy is a strategy that involves introduction of a viral or a bacterial gene into tumor cells resulting in the conversion of a non-toxic compound into lethal active molecules capable of inducing tumor cell death [18]. A critical factor in this strategy is that the gene encodes an enzyme which converts a prodrug into a cytotoxic drug. More importantly, this strategy is based on evidence that prodrug-activating enzymes are normally absent or expressed at low levels in mammalian cells [19]. Consequently the tumor-targeting viral vector is necessary to restrict enzyme expression to the transduced tumor cells. Several suicide gene therapies have been evaluated using adenoviral, retroviral, or non-viral vector delivery methods in numerous clinical trials [17, 20–24]. The most widely investigated suicide gene therapies against GBM are Herpes Simplex Virus Thymidine Kinase (HSV-TK) gene therapy and Cytosine Deaminase 5-fluorocytosine (CD/5-FC) [13, 17, 25–27].

**Figure 1:**
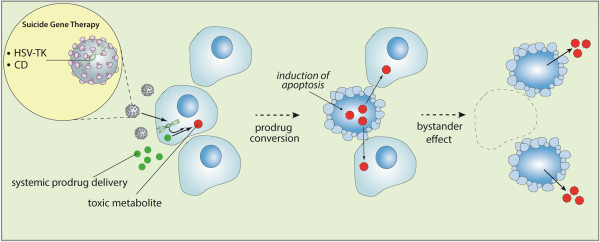
**Strategy for suicide gene therapy.** The aim of suicide gene therapy strategy is to increase the delivery of toxic metabolites to tumor cells and result in efficient cell death. Initially, a gene encoding a prodrug-activating enzyme is delivered by a tumor-targetting viral vector. Subsequent systemic administration of an inactive prodrug results in generation of a toxic metabolite and cell death of the transduced cells and non-trasduced bystander tumor cells (bystander effect) only at the tumor site.

The possibility of using HSV-TK as gene therapy was first reported by Moolten in 1986 [28]. HSV-TK can catalyze the phosphorylation of nucleoside analogues such as ganciclovir (GCV: a synthetic analogue of 2′-deoxy-guanosine) which is a poor substrate for the mammalian TK. Following the systemic administration of the inactive prodrug, GCV is converted by HSV-TK into a toxic metabolite called GCV-triphosphate which is incorporated into the DNA of actively proliferating cells (Figure 1). GCV-triphosphate consequently blocks DNA replication and inhibits cell division [28, 29]. Apoptosis underlies the mechanism of cytotoxicity induced by the HSV-TK/GCV gene therapy [30]. The HSV-TK gene therapy is cell cycle dependent. Therefore, one of the advantages of this therapy is that it exhibits selective cytotoxicity to only actively dividing cells transduced with HSV-TK. The other advantage is the so-called bystander effect where toxicity is transferred directly from infected cells to adjacent non-infected cells thereby enhancing the treatment effect [31]. A possible mechanism that can account for this effect is that non-transduced cells are killed by the spread of phosphorylated nucleoside analogues through gap-junctions, facilitated by cell-to-cell contacts [15, 32–34]. A second possible mechanism is the accumulation of phosphorylated nucleoside analogues in neighboring cells inducing apoptosis of non-transduced cells [35, 36]. A third mechanism may involve phagocytosis induced by neighboring transduced cells as a result of apoptotic vesicle formation. Several cellular components could be shared with the neighboring cell during this process resulting in the delivery of an apoptotic signal [37].

HSV-TK/GCV gene therapy has demonstrated promising results in animal models [12, 38]. Recently, these results have prompted clinicians to examine HSV-TK/GCV gene therapy for glioblastoma. Multiple Phase I and II clinical trials demonstrated that HSV-TK/GCV gene therapy is a relatively safe strategy [21, 39]. There is some evidence, however, suggesting that HSV-TK transduced cells may become resistant to the prodrug, therefore requiring combination of suicide gene therapy with chemo-radiotherapy [25, 40–42]. One of the largest randomized Phase III clinical trials was conducted by Rainov, where 248 patients with newly diagnosed, previously untreated GBM, were randomized into a control group (surgical resection and radiotherapy) or gene therapy group (surgical resection and radiotherapy plus adjuvant replication-competent retrovirus mediated HSV-TK/GCV gene therapy during surgery). Although this trial proves that HSV-TK gene therapy was safe, there was no difference in median survival and tumor progression between groups. Unfortunately, based on these data, there was no therapeutic benefit of retrovirus mediated HSV-TK gene therapy. The authors concluded that improved high efficiency delivery and distribution strategy of therapeutic genes is likely necessary [43]. Despite promising results *in vitro* and *in vivo*, the anti-tumor effect of retrovirus mediated HSV-TK gene therapy in clinical trials remains insufficient due to very low transfection efficiency. In support of this, Sandmair *et al.* reported a Phase I clinical trial of 21 patients diagnosed with primary or recurrent GBM who were treated with replication-defective retrovirus or adenovirus. The results of this study showed that the median survival of the adenovirus mediated HSV-TK/GCV gene therapy group was significantly longer than the retrovirus mediated group [22]. Germano *et al.* also reported the safety of replication-defective adenoviral-mediated HSV-TK gene therapy in a Phase I trial, where the average survival of the treatment group was 112.3 weeks and one patient survived as long as 248 weeks from diagnosis. Collectively these trials indicate that using adenovirus to deliver the HSV-TK gene may be better in contrast to retroviral vectors [21]. At least 10% transfection efficiency is needed in order to obtain a significant reduction in tumor volume based on the results of the rat glioma xenograft experiments [44]. Clinical trials utilizing adenoviral delivery suicide gene therapy for glioma are currently underway (Table 1).

**Table 1:** **Ongoing clinical trials for gene therapy of GBM**

Type of gene therapy	Phase	Vector	Gene	Prodrug	Nation	ID	Title
Suicide	Pilot	NSC	CD	5-FC	USA	NCT01172964	A Pilot Feasibility Study of Oral 5-Fluorocytosine and Genetically-Modified Neural Stem Cells Expressing E. Coli Cytosine Deaminase for Treatment of Recurrent High Grade Gliomas
Suicide	I	AV	HSV-TK	Valacyclovir	USA	NCT00751270	Phase 1b Study of AdV-tk + Valacyclovir Combined With Radiation Therapy for Malignant Gliomas
Suicide/immune-mediated	I	AV	HSV-TK	Valacyclovir	USA	NCT01811992	Combined Cytotoxic and Immune-Stimulatory Therapy for Glioma
		AV	Flt3L	**-**			
Suicide	I	RV (Toca 511)	CD	5-FC	USA	NCT01470794	Study of a Retroviral Replicating Vector to Treat Patients Undergoing Surgery for a Recurrent Malignant Brain Tumor
Suicide	I	RV (Toca 511)	CD	5-FC	USA	NCT01985256	Study of a Retroviral Replicating Vector Given Intravenously to Patients Undergoing Surgery for Recurrent Brain Tumor
Suicide	I	AV	HSV-TK	Valacyclovir	USA	NCT00634231	A Phase I Study of AdV-tk + Prodrug Therapy in Combination With Radiation Therapy for Pediatric Brain Tumors
Suicide	I/II	RV (Toca 511)	CD	5-FC	USA	NCT01156584	A Study of a Retroviral Replicating Vector Administered to Subjects With Recurrent Malignant Glioma
Suicide	II	AV	HSV-TK	Valacyclovir	USA	NCT00589875	Phase 2a Study of AdV-tk With Standard Radiation Therapy for Malignant Glioma (BrTK02)
Oncolytic	I	HSV (HSV1716)	**-**	**-**	USA	NCT02031965	Oncolytic HSV-1716 in Treating Younger Patients With Refractory or Recurrent High Grade Glioma That Can Be Removed By Surgery
Oncolytic	I	MV	CEA	**-**	USA	NCT00390299	Viral Therapy in Treating Patients With Recurrent Glioblastoma Multiforme
Oncolytic	I	AV (DNX-2401)	**-**	**-**	Spain	NCT01956734	Virus DNX2401 and Temozolomide in Recurrent Glioblastoma
Oncolytic	I	PoV (PVS-RIPO)	**-**	**-**	USA	NCT01491893	Poliovirus Vaccine for Recurrent Glioblastoma Multiforme (GBM)
Oncolytic	I	AV (DNX-2401)	**-**	**-**	USA	NCT00805376	DNX-2401 (Formerly Known as Delta-24-RGD-4C) for Recurrent Malignant Gliomas
Oncolytic	I/II	HSV (G47Delta)	LacZ	**-**	Japan	JPRN-UMIN000002661	A Clinical Study of a Replication-Competent, Recombinant Herpes Simplex Virus Type 1 (G47delta) in Patients With Progressive Glioblastoma
Oncolytic	I/II	PaV (H-1 PV)	**-**	**-**	Germany	NCT01301430	Parvovirus H-1 (ParvOryx) in Patients With Progressive Primary or Recurrent Glioblastoma Multiforme
Oncolytic	I/II	AV (Delta24-RGD)	**-**	**-**	Netherlands	NCT01582516	Safety Study of Replication-competent Adenovirus (Delta-24-rgd) in Patients With Recurrent Glioblastoma
Oncolytic	I/II	AV (Delta24-RGD)	**-**	**-**	Netherlands	EUCTR2007-001104-21-NL	A Phase I/II Trial of a Conditionally Replication-Competent Adenovirus (delta-24-rgd) Administered Convection Enhaced Delivery in Patients With Recurrent Glioblastoma Multiforme
*Suicide	I	NSC	CD	5-FC	USA	NCT02015819	Genetically Modified Neural Stem Cells, Flucytosine, and Leucovorin Calcium in Treating Patients With Recurrent High-Grade Gliomas
*Oncolytic	I/II	NDV	**-**	**-**	Israel	NCT01174537	New Castle Disease Virus (NDV) in Glioblastoma Multiforme (GBM), Sarcoma and Neuroblastoma
*Oncolytic/immune-mediated	I	HSV (M032)	IL-2	**-**	USA	NCT02062827	Genetically Engineered HSV-1 Phase 1 Study

A search was conducted on a publically available online database made available by the U.S. National Institutes of Health (http://clinicaltrials.gov/) and International Clinical Trials Registry Platform (http://apps.who.int/trialsearch/Default.aspx) as of February 2014. *Abbreviations*: *5-FC*, 5-fluorocytosine; *AV*, adenovirus; *CEA*, carcinogenic embryonic antigen; *CD*, cytosine deaminase; *Flt3L*, fms-like tyrosine kinase-3 ligand; *HSV*, herpes simplex virus; *IL*, interleukin; *MV*, measles virus; *NDV*, new castle disease virus; *NSC*, neural stem cell; *PaV*, parvovirus; *PoV*, poliovirus; *RV*, retrovirus; *TK*, thymidine kinase; *, registered but not yet recruiting.

Suicide gene therapy using cytosine deaminase/5-fluorocytosine (CD/5-FC) has been an extensively studied form of anti-glioma gene therapy [17]. CD is a microbial or a yeast enzyme capable of converting an effective anti-fungal drug, 5-FC, to the highly toxic anti-cancer compound 5-fluorouracil (5-FU) [45, 46]. Since CD is absent in mammalian cells, 5-FC has minimal human toxicity. The toxic effects of 5-FU are mediated by the conversion of 5-FU to 5-FU triphosphate which interferes with RNA processing after incorporation into RNA; and to 5-fluoro-2′-deoxyuridine 5′-monophosphate, which irreversibly inhibits thymidylate synthase, and blocks DNA synthesis (Figure 1) [19]. Similar to HSV-TK gene therapy, apoptosis underlies the cytotoxic mechanism of CD/5-FC gene therapy [30, 47]. 5-FU is a small molecule that can diffuse in and out of transduced and neighboring cells, resulting in significant bystander effects which do not require cell-cell contact and functional gap junctions [48, 49]. In comparison to HSV-TK gene therapy, CD/5-FC gene therapy demonstrated a greater anti-tumor effect when observed in a colorectal xenograft tumor model where only 4% of tumor cells are transduced [50]. Dong *et al.* reported that replication-deficient adenovirus vectors carrying the *CD* gene and subsequent administration of 5-FC resulted in significant prolonged survival in glioma-bearing rats [51]. Several attempts have been made to increase the efficacy of CD/5-FC gene therapy. Adachi *et al*. reported that a second enzyme, uracil phosphoribosyltransferase (UPRT), which is absent from mammalian cells, directly converts 5-FU into 5-fluorouridine monophosphate which enhances the cytotoxicity of CD/5-FC gene therapy in an experimental malignant brain tumor, suggesting that co-expression of *CD* and *UPRT* genes have synergistic anti-tumor effects [52]. In addition, the combination of CD/5-FC and UPRT gene therapy also enhances conventional radiotherapy in an animal model of glioma [53]. Further enhancement of cytotoxicity is accomplished by using replication-defective adenoviral vector encoding a mutant bacterial *CD* gene with increased affinity for 5-FC [19]. The combination of this recombinant *CD* gene with ionizing radiotherapy has shown significant tumor cell killing and inhibition of tumor growth in glioma xenograft models [19]. Recently, a second generation non-lytic retroviral replicating vector (Toca 511) demonstrated stable delivery of CD resulting in significant survival benefit without treatment related toxicity in a mouse glioma model [54]. Furthermore, synergistic therapeutic efficacy of TMZ, which is the most frequently used treatment for patients with GBM, was observed in combination with Toca 511 with subsequent administration of 5-FC resulting in a survival advantage in mice bearing TMZ-sensitive glioma [55].

A novel approach to suicide gene therapy involves the use of genetically engineered neural stem cells as a vector. Neural stem cells have the ability of continuous proliferation and differentiation into neuronal or glial cells [56–58]. One of the great advantages of using neural stem cells as a vector for GBM therapy is their invasive capability directed towards tumor cells, even when injected adjacent to the tumor [59, 60]. Genetically engineered neural stem cells have been successfully used to deliver CD and HSV/TK gene products into GBM [59, 61, 62].

CD/5-FC gene therapy has reached clinical trials and Toca 511 or genetically modified neural stem cells used as vectors are currently under investigation in patients with recurrent high grade glioma [54, 55] (Table 1). An additional type of stem cell vector is the mesenchymal stem cell. Mesenchymal stem cells are non-hematopoietic, multipotent stem cells. In comparison to neural stem cells, mesenchymal stem cells have advantages because they are easily acquired from patient tissues such as bone marrow, adipose tissue, muscle tissue, and peripheral blood stream [27, 63]. Their intrinsic ability to migrate to the site of injury and inflammation allows them to invade into tumors [64]. Owing to this strong homing behavior, mesenchymal cells have been used as a vector for gene therapy against glioma [65]. Some suicide gene therapies have employed mesenchymal stem cells as a vector, including HSV/TK, CD, and HSV/TK combined with connexin-43 to enhance bystander effect [66–68].

#### Oncolytic gene therapy

**Figure 2:**
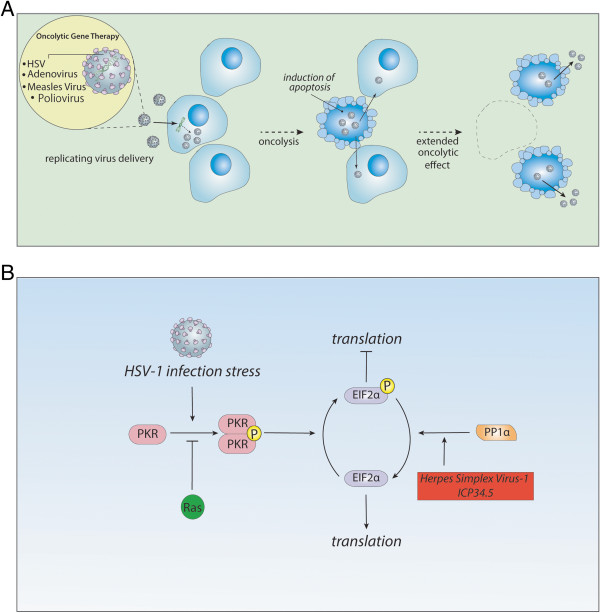
**Strategy and mechanism for oncolytic gene therapy. (A)**; Oncolytic gene therapy employs replication-competent virus vectors capable of selective replication in target tumor cells. Spreading to new adjacent progeny cells occurs as the host cell is lysed and progeny virus is released. **(B)**; Most viruses can replicate poorly in normal cells by a defense mechanism as follows. In response to viral infection, Protein Kinase R (PKR) in the host cells shut off protein synthesis by which PKR dimerizes and is inactivated by autophosphorylation resulting in the conversion of eukaryotic initiation factor-2 alpha (EIF-2α) into its inactive state following phosphorylation, which is required for translation initiation. Consequently, translation is arrested in the infected host cells as an anti-viral protective mechanism. However, the ICP34.5 in HSV-1 can overcome this defense by recruiting protein phosphatase-1α to dephosphorylate EIF-2α allowing protein synthesis to proceed. Therefore, when a deletion of *γ34.5* gene is engineered, the HSV-1 mutant can no longer successfully proliferate in non-replicating cells. HSV-1 lacking ICP34.5 activity can only infect cells with defective PKR pathway. In tumor cells, PKR autophosphorylation is blocked due to Ras activation, permitting replication of viruses lacking the *γ34.5* gene in tumor cells with hyper-activated Ras.

A substantial focus in viral vector development has been the creation of genetically engineered adenoviruses and retroviruses. However, researchers are confronted with the limitations of their low efficiency for distribution, delivery to target cells and difficulties in achieving prolonged efficacy. Oncolytic gene therapy employs replication-competent viral vectors in order to increase the toxicity and efficiency against the tumor. Oncolytic viral vectors have the ability to selectively replicate in target tumor cells, and then to release viral particles and to spread to new adjacent progeny cells as the host cell is lysed (Figure 2A). Although, in some studies, immunosuppression has been shown to improve viral oncolytic effect, there is increasing evidence that oncolytic gene therapy will ultimately require an antitumor immune response as well as disruption of the tumor microenvironment such as inhibition of angiogenesis [10, 69–74]. The transduction efficiency of replication-competent viral vectors in tumors is significantly higher than that of replication-deficient viral vectors [17]. Oncolytic HSV, conditionally replicating adenovirus, measles virus (MV), poliovirus (PoV), Newcastle disease virus, parvovirus (H1-PV), and reovirus have all been employed and clinically tested in oncolytic gene therapy strategies for GBM (Table 1) [17, 27, 75]. Below, we describe the strategies for anti-glioma oncolytic viral therapy.

HSV-1 is an enveloped double strand DNA virus with neurotropic properties capable of replication in dividing and non-dividing cells [13, 76, 77]. Wild type HSV-1 may either proceed to a lytic life cycle or stay in an intranuclear episome which is never integrated into the host genome. An advantage of an HSV-1 mediated approach is the documented sensitivity to acyclovir and GCV, thus adding to its safety profile. G207 is a conditionally replicating HSV mutant vector that is a genetically engineered HSV-1 (F) strain lacking the genes necessary for viral replication in normal cells. G207 has a deletion in both copies of *γ34.5* gene which encodes for the protein synthesis promoting factor infected cell protein (ICP) 34.5. In addition, the *Unique Long* (*UL*) *39* gene is inactivated by an insertion of the *LacZ* reporter gene into the region of the *UL39* gene encoding ICP6, thereby disrupting the large subunit of the enzyme ribonucleotide reductase (RR) [78]. The enzyme, RR, is crucial for nucleotide synthesis after infection of post-mitotic cells such as neurons [79, 80]. Oncolytic HSV virus which has an impaired RR cannot replicate in non-dividing cells but can replicate only in dividing cells, which is due to the mitotic cells providing cellular RR and circumventing the need for viral RR. Therefore, oncolytic viruses which have mutation in *UL39* gene can achieve effective lytic activity only in dividing cells such as malignant glioma. However, it was estimated that only 5-15% of malignant glioma cells are in mitotic phase at any moment in time, meaning that the majority of malignant glioma cells can escape this type of oncolytic gene therapy [81, 82]. However, the ICP6-defective HSV retains significant replicative ability in p16-deficient cells independent of cell cycle status [81]. Therefore, G207 can target non-dividing cells with p16 deletion or inactivation [81].

An added safety advantage of the RR-deficient virus, G207, is modest hypersensitivity to anti-viral agents such as acyclovir and GCV [83]. The bacterial reporter gene *LacZ* is a useful histological marker to monitor the distribution of viral infection within the target tissues [84]. The neurovirulence gene, *γ34.5* is crucial to overcome host cell defense mechanisms after infection. During viral infection, a stress response is initiated by the host cells. Protein Kinase R (PKR) activation arrests translation in the infected host cells in order to induce an anti-viral protective mechanism by phosphorylating and inactivating eukaryotic initiation factor-2 alpha (EIF-2α) which is required for the initiation of translation. However, ICP34.5 in HSV-1 recruits protein phosphatase-1α to dephosphorylate EIF-2α and promote protein synthesis (Figure 2B) [85]. HSV-1 lacking ICP34.5 activity can only infect cells with a defective PKR pathway [86]. In tumor cells, PKR autophosphorylation is blocked due to Ras activation; therefore it allows selective replication of the viruses lacking the *γ34.5* gene in tumor cells with hyper-activated Ras (Figure 2B) [84, 87–89]. *UL39* gene encoding RR is crucial for virus replication given its functional role in catalyzing the formation of ribonucleotides, which are essential to nucleotide synthesis from ribonucleotides. Therefore RR is critical for viral DNA synthesis. Virus replication depends on RR in normal non-dividing cells; therefore the lack of viral RR expression in the G207 is compensated for by the tumor cells with high RR activity which results in highly specific targeting of tumor cells.

Preclinical studies using G207 demonstrated decreased tumor growth in experimental gliomas and a high safety profile [78, 90–93]. Based on these results, G207 mediated oncolytic therapy was taken to clinical trials to begin evaluating its efficacy in anti-glioma therapy. Markert *et al.* reported a Phase I clinical trial using G207 in 2000 [94]. In total, 21 patients with progressive malignant glioma were enrolled. The trial demonstrated no treatment-related toxicity or serious adverse events; and no evidence of HSV encephalitis was observed. A positive therapeutic response was identified in eight patients, and one patient survived 5.5 years after the treatment. A maximally tolerated dose could not be determined because there were no dose-limiting toxicity even with inoculation of 3 × 10^9^ Plaque-Forming Units (PFUs) [94]. In a Phase Ib study, six patients with recurrent glioma received G207 inoculation totaling 1.15 × 10^9^ PFUs both prior to surgery and post operatively. The virus was injected either directly into the tumor or into the resected tumor cavity using a stereotactically guided catheter. Three of the patients showed subsequent improvement and the overall survival was greater than six months. In this study, there was no evidence of virus-related toxicities and G207 gene therapy was shown to have an excellent safety profile for repeated dose delivery as well as direct injection into the resected tumor cavity [95].

Another genetically engineered HSV-1 mutant is HSV1716, derived from the parent wild-type strain HSV-1 17+ [96]. This conditionally replicative mutant virus has a 759 bp deletion in both copies of *γ34.5* gene, inactivating the ICP34.5 protein and the intact *UL39* gene. HSV1716 is capable of replication in dividing cells but not differentiated cells, conveying tumor cell selectivity similar to G207. In 1994, Valyi-Nagy *et al.* demonstrated that HSV1716 was nonvirulent in severe combined immunodeficiency mice making it a promising candidate for oncolytic gene therapy [97]. Moreover, the studies in animal models supported pursuing HSV1716 as a potential novel treatment strategy for malignant brain tumors [98–100]. The promising safety and efficacy profiles allowed HSV1716 to be tested for anti-glioma therapy in humans. As with G207, the safety and toxicity of HSV1716 administration in patients was first demonstrated in a Phase I clinical trial consisting of nine patients with recurrent glioma that received stereotactic intratumoral injections. Prior to the start of therapy, all patients had undergone surgery and radiotherapy and six patients received chemotherapy. Four patients remained alive for 14–24 months after the virus administration. None of the patients demonstrated signs of encephalitis following treatment, and there was no need to use anti-herpetic medication. It was remarkable that no major neurological manifestation was noted. The treatment decelerated tumor growth and increased the survival to three years in one patient and to four years in two patients. In this trial, a dose escalation study was also carried out with the initial doses of 1× 10^3^ to 1 × 10^5^ PFUs. A maximum tolerated dose was not determined because the highest dose used in this study showed no adverse effects [101].

In a subsequent Phase Ib trial, Papanastassiou *et al.* demonstrated replication activity of HSV1716 within the tumors. All patients enrolled in this trial were inoculated with 1 × 10^5^ PFUs intratumorally, and then underwent tumor resection four to nine days after injection. The resected tumors were analyzed for viral replication activity. While no treatment related toxicity was observed, there was evidence of viral replication upon histological examination [102]. A third clinical study was reported by Harrow in 2004 [103]. In this study, 12 patients with recurrent or newly diagnosed GBM underwent surgical resection. HSV1716 was injected into multiple sites around the resection cavity and none of the patients showed viral injection-related toxicity. Three patients demonstrated clinically stable disease and increased survival following surgery and viral injection at 15, 18, and 22 months [103]. HSV1716 has also been clinically used for advanced melanoma [10, 104]. In three patients receiving multiple intranodular injections, immunohistochemical staining of injected nodules revealed evidence of virus replication confined to tumour cells without toxic effects [105]. Taken together, these studies show that HSV1716 has been successfully used in clinical trials for glioma therapy as well as other types of cancer. However, one drawback of these models is the required deletion of *γ34.5* genes which reduces the viral replication ability, and limits its efficacy [106].

Oncolytic HSVs which have deletions in both copies of *γ34.5* genes have limited or no replication in GSCs [107]. G47Delta is the new generation of oncolytic HSV. In order to restore GSC sensitivity, G47Delta has an additional deletion of the gene encoding ICP47 resulting in enhanced major histocompatibility complex (MHC) class I antigen presentation and enhanced immune response [107, 108]. In addition, this deletion causes promoter shift for the *unique short 11* gene which blocks the effect of interferons and consequently to increase virus replication in tumor cells [108, 109]. G47Delta is being examined in pre-clinical models and preparing to proceed to clinical trials [108, 110–112]. A Phase I/II clinical trial using G47Delta which further enhances specificity and safety is currently underway evaluating the efficacy and safety for recurrent or progressive GBM (Table 1).

AV are nonenveloped viruses with double strand DNA capable of infecting both proliferating and quiescent cells. AV vectors enter central nervous system (CNS) cells by receptor-mediated endocytosis; however, they are not integrated into cellular DNA, but exist as episomes in which AV’s DNA is transcribed and translated using the intrinsic cellular machinery [113]. Given that AV’s are not inserted into the host genome, there is minimal risk of insertional mutagenesis. Two commonly investigated conditionally replicating AVs in glioma are ONYX-015 (also known as dl1520) and Ad5Delta24, both of which target cells with impaired signaling pathways [17, 27].

ONYX-015 has a deletion in the 55kD protein early region 1B-55kD (E1B-55kD) which conveys a selective phenotype to it [114]. E1B-55kD normally binds and inactivates the tumor suppressor p53 preventing the induction of p53 apoptosis in the infected cell before the virus cycle is complete. Consequently, the cells with functional p53 protein cannot assist viral replication without E1B-55kD. The absence of E1B-55kD results in selective replication in p53-deficient tumor cells [114–116]. An important consideration is that some studies have identified that there are additional beneficial mechanisms that may provide oncolytic activity of ONYX-015 in gliomas which are p53-independent, and may even be increased in p53 intact glioma cells [117–120]. Preclinical studies of ONYX-015 in human malignant glioma xenografts derived from primary tumors demonstrated a high anti-tumor activity and widespread intratumoral replication in p53 wild-type tumors as well as p53-mutant tumors [117]. Furthermore, the efficacy of oncolytic therapy of the virus was enhanced by radiation therapy [121]. ONYX-015 is a promising agent for further anti-glioma research in animal model studies, and ONYX-015 needs to be tested in clinical trials for malignant glioma [117]. The initial Phase I dose-escalation clinical study in brain tumors was conducted in 24 patients with recurrent malignant glioma who were subdivided into four groups of six patients. Each patient received ONYX-015 at a dose from 1 × 10^7^ PFUs to 1 × 10^10^ PFUs injected into 10 different sites within the tumor bed cavity following surgical resection. None of the patients showed serious adverse effects related to ONYX-015; however, the maximum tolerated dose was not reached, even at the highest dose of 1 × 10^10^ PFUs. The median time to progression and the median survival time were 46 days and 6.2 months, respectively. Despite ONYX-015′s promising safety profile, there was no observable significant therapeutic benefit [122]. ONYX-015 was also used in clinical trials for other types of cancer such as squamous cell carcinoma and hepatobiliary carcinoma [10]. In contrast to the clinical trial for glioma, they showed anticancer effect without significant adverse effects [10, 123–125]. These results suggest that ONYX-015 could be applied more effectively to patients with an earlier stage of GBM.

The Ad5Delta24 has a 24 bp deletion in the viral protein early region 1A. This deletion inhibits virus function by interfering with the retinoblastoma (Rb) protein. Normally, phosphorylated Rb inhibits cell proliferation by binding and inactivating the transcription factor E2F. Infected cells with wild-type AV are transformed by inhibiting Rb-hypoP (hypo-phosphorylated form of Rb) in the proliferative phase of the cell cycle (G1 to S cell phase). Rb is a tumor suppressor protein that functions in a pathway that is commonly altered in gliomas [126, 127]. In this manner, Ad5Delta24 can selectively replicate in glioma cells with an impaired Rb pathway [128]. Preclinical studies using the Ad5Delta24 demonstrated significant growth inhibition of glioma xenografts in nude mice. However, normal fibroblasts or cancer cells with intact Rb activity were refractory to the viral therapy [128]. The Ad5Delta24 was further genetically modified by the insertion of the integrin-binding Arg-Gly-Asp (RGD) motif into the fiber knob domain of the viral fiber protein, enabling anchoring to integrins to enhance viral tropisms (Ad5Delta24-RGD) [129]. Preclinical studies using this vector with malignant glioma cell lines successfully demonstrated that Ad5Delta24-RGD enhanced the specific targeting of the tumor cells and increased the oncolytic efficacy against glioma. Furthermore, the survival of mice harboring glioma xenografts that received an intratumoral injection of Ad5Delta24-RGD was improved in comparison to the mice that received Ad5Delta24 control vector [130]. An additional preclinical study in glioma xenografts demonstrated that the oncolytic activity of Ad5Delta24-RGD was enhanced by irradiation [131]. A Phase I and I/II clinical trial using Ad5Delta24-RGD (DNX-2401) is currently underway evaluating efficacy for recurrent malignant glioma (Table 1) [17, 27, 132].

MV, well-known as a human pathogen, is a single-stranded, enveloped RNA virus belonging to the family *Paramyxoviridae*. MV is known to be neurotropic and rarely causes encephalitis. MV’s entry into cells requires attachment of the hemagglutinin envelope glycoprotein H to its cellular receptors such as CD46 and Signaling Lymphocyte Activating Molecule followed by fusion with the cell membrane through the envelope fusion glycoprotein F. These two glycoproteins play an important role for oncolytic specificity and efficacy. Mutations in the H protein of the attenuated MV, known as the Edmonston strain (MV-Edm), has a high affinity for cellular CD46 receptors which are ubiquitously overexpressed in a wide range of tumors [133–136]. The F protein is responsible for membrane fusion and induces the formation of multinucleated syncytia followed by apoptosis [137]. The MV-Edm was genetically engineered to express the circulating carcinogenic embryonic antigen (CEA) in order to monitor the viral activity and maintenance of MV [138, 139]. This MV-CEA demonstrated favorable anti-tumor activity and safety profile in animal models including a primary tumor GBM xenograft model [75]. Based on these results, a Phase I clinical trial of the MV-CEA strain in patients with GBM is currently underway (Table 1) [17, 27]. MV variants have been engineered to express either interleukin (IL)-13 as a ligand to the GBM specific receptor IL-13Rα2 or a single-chain antibody against the vIII deletion variant of EGFR [140–142]. These MV variants have the capability of targeting specific proteins expressed on glioma cells thereby increasing their oncolytic activity and specificity.

PoV is a positive-sense, non-enveloped RNA virus with natural neurotropism belonging to the *Picornaviridae* family and is the virus responsible for human poliomyelitis [136]. Viral neurotropism is attributed to the viral entry receptor CD155 (also known as the poliovirus receptor) which has been ectopically expressed and evaluated in malignant glioma cells [143–145]. Viral neuropathogenicity is attributed to an internal ribosomal entry site (IRES), located in the 5′ untranslated region of the poliovirus genome, which mediates viral protein translation [146]. In order to eliminate the neurotoxicity, Gromeier et al. created a recombinant intergeneric virus by replacing the PoV IRES sequence with a nonpathogenic version from human rhinovirus type 2 [147]. This recombinant PoV, PVS-RIPO, is derived from the Sabin polio vaccine, which has greatly diminished viral proliferation in normal neuron cells while retaining significant lytic growth in malignant glioma cells [143]. When delivered directly into the spinal cord, PVS-RIPO significantly diminished poliomyelitis-like neurotoxicity in both mice transgenic for the CD155 receptor and nonhuman primates [143, 144]. Preclinical studies of PVS-RIPO demonstrated significant biologic effects. Treatment of athymic mice bearing intracerebral glioma xenografts with PVS-RIPO attenuated tumor progression and lead to tumor elimination [143]. Based on these results, a Phase I clinical trial using PV-RIPO is currently underway for application in recurrent malignant glioma (Table 1).

Stem cells used as carriers of oncolytic viruses have become a promising approach because of their natural tropism and ability to infiltrate solid tumors so that they can deliver the viruses at further distance within large lesions [148]. There have been several examples demonstrating the usefulness of using stem cells (neural stem cells or mesenchymal stem cells) to deliver conditionally replicating HSV and AV in preclinical studies [149–152]. As expected, the reach of viral delivery was strongly enhanced by using neural stem cells. Mesenchymal stem cells carrying conditionally replicating AV has demonstrated that the stem cell vector can suppress the immune response against the virus, which makes it possible to prolong viral activity [152]. Intra-arterial delivery of mesenchymal stem cells carrying Ad5Delta24-RGD selectively localized to human gliomas and were capable of delivering and releasing Ad5Delta24-RGD into the tumor, resulting in improved survival and tumor eradication in glioma xenograft mouse models [153]. These results suggest that stem cells carrying oncolytic virus may be administered systemically and demonstrate viral delivery more efficiently.

#### Cytokine mediated gene therapy

**Figure 3:**
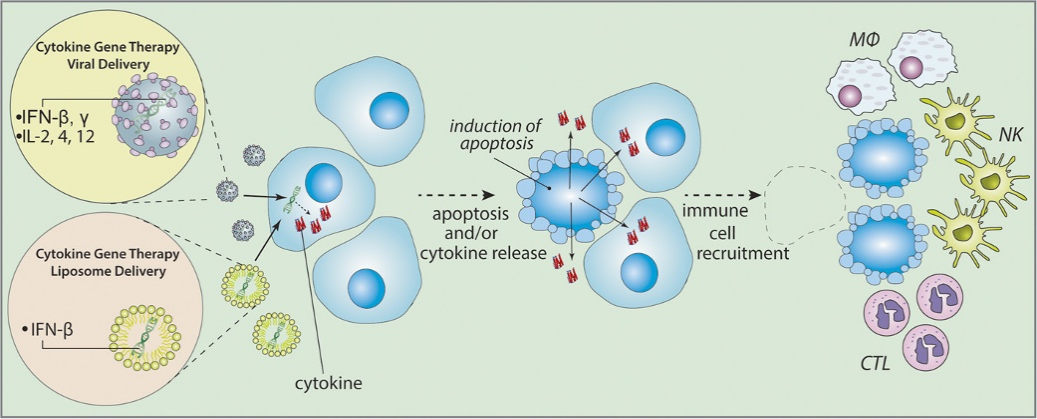
**Strategy of cytokine mediated gene therapy.** Cytokine mediated gene therapy involves tumor-selective gene transfer and in situ expression of various cytokine genes such as interleukin (IL) and interferon (IFN) capable of attracting immunocompetent cells such as macrophages (MΦ), natural killer cells (NK), and cytotoxic T lymphocytes (CTL) inducing immune response.

The underlying principle of cytokine mediated gene therapy involves tumor-selective gene transfer and in situ expression of various cytokine genes such as IL-2, −4, −12, and interferon (IFN)-β, −γ which can induce robust immune responses restricted to antigens specific to glioma cells [17, 37, 77, 154]. Given that the CNS is relatively isolated from the systemic immune system, gliomas can effectively evade the host immune response [13, 27]. Several physiological mechanisms unique to the CNS immune system include lack of antigen-presenting cells such as dendritic cells (DCs); lack of production of anti-inflammatory mediators such as transforming growth factor (TGF)-β; weaker expression of major MHC class I and II; and the existence of BBB. These mechanisms play important roles to protect the CNS from immunological attack (Figure 3). Therefore, it is challenging to stimulate the immune system to develop an effective anti-tumor response against gliomas [69]. Indeed, various cytokines such as IFNs and IL have been used clinically for the treatment of cancer, including chronic myeloid leukemia, T cell and B cell lymphomas, melanoma, and renal carcinoma [155, 156]. Several preclinical studies have demonstrated the feasibility of mobilizing the immune response against glioma cells. The susceptibility of glioma stem cells to the cytotoxic effects of the immune system provide the basis for developing anti-glioma immune gene therapy based on immunomodulation. Melanoma cells infected with a viral vector carrying the gene for granulocyte-macrophage colony stimulating factor were injected subcutaneously in mouse. This cytokine generated a strong immune response against intracranial melanoma in the mouse model [157, 158]. With regard to the treatment for glioma, using various types of vectors for cytokine gene delivery may allow local augmentation of immune response. Since then, a variety of cytokines such as interleukin and interferon gene therapies have been studied to activate and enhance the effectiveness of immune therapy against tumors.

IFN-β, type I interferon genes, are primarily produced by specialize antigen presenting cells such as DCs post viral infection [155]. IFN-β is an immunostimulatory molecule inducing MHC class I expression, leading to an increase in cytotoxic T cells activity, enhancing the generation of T helper cells and activating natural killer (NK) cells, DCs, and inducing macrophage activity [154, 155, 159, 160]. These immunomodulatory activities can lead to a robust anti-tumor immune response. Qin *et al.* reported a successful effect following *IFN-β* gene delivery using a replication-deficient adenovirus under the control of a cytomegalovirus (CMV) promoter [160]. Direct injection of the *IFN-β* gene with a replication-deficient adenovirus demonstrated tumor regression in human xenografts including glioma, through the activation of NK cells. Survival was significantly prolonged in these mice [161]. Based on these results, an IFN-β-expressing replication-defective AV was used in Phase I clinical trials for recurrent malignant glioma. The 11 enrolled patients received stereotactic injection of the vector into the tumor and surrounding intact brain following resection. One out of 11 patients experienced treatment related dose-limiting toxicity. Tumor histopathological analysis revealed dose-related induction of apoptosis and necrosis [162].

The use of a different type of vector, cationic liposomes, was also used for *IFN-β* gene transfer. Local administration of cationic liposomes containing the *IFN-β* gene induced marked inhibition of tumor growth, NK cell activation, and prolonged survival of mice bearing human glioma xenografts [163, 164]. In addition, cationic liposomes containing the *IFN-β* gene induced cytotoxic T-cell immunity against mouse glioma cells and marked tumor growth inhibition by activation of the immune response in experimental gliomas [165, 166]. Based on these results, this strategy reached a Phase I clinical trial for recurrent malignant glioma. There was no observable toxicity attributable directly to the use of liposome. Two patients with anaplastic astrocytoma experienced more than 50% tumor reduction for at least 16 months [167]. Subsequent histological examination demonstrated a dramatic change in the tumor tissue including the presence of CD8 positive lymphocytes and marked macrophage infiltration as well as apoptosis and neovascularization [168]. The addition of IFN-β to TMZ chemotherapy for patients with newly diagnosed GBM results in a more favorable outcome than standard therapy of TMZ alone [155, 169]. Neural stem cells have also been used to deliver a combination of CD and IFN-β together to enhance the bystander effect and the immune response against the glioma, demonstrating improved anti-tumor response compared to CD alone [24].

Similar to IFN-β, the type II interferon gene, IFN-γ, is also an immune stimulatory cytokine produced by NK cells, dendritic cells, and activated T-lymphocytes [69, 170]. IFN-γ inhibits the adhesion of malignant glioma cells *in vitro* and diminishes the invasive phenotype of glioma cells by reducing binding to extracellular matrix macromolecules [171]. Single-agent activity of IFN-γ has appeared to be less effective than IFN-β [154] and several attempts to focus on the use of combination regimens have been performed. The use of AVs expressing tumor necrosis factor (TNF)-α or IFN-γ introduced into tumors enhanced infiltration of CD4 and CD8 positive T cells in addition to increasing expression of MHC class I and II on the tumor cells in a mouse glioma model. Intracranial administration of both vectors led to a statistically significant increase in survival of tumor bearing mice [172]. In addition, simultaneous delivery of IFN-γ inducible protein 10 and TNF-α, both potent immune-stimulatory cytokines, was attempted in a GBM mouse model using recombinant parvovirus [173]. The study demonstrated synergistic activity when using both vectors and complete regression of tumors generated from murine glioma cells that had been infected *in vitro* with both cytokines before implantation. Although IFN-γ is thought to have anti-angiogenic properties, cytokine-mediated immune stimulation was likely responsible for the therapeutic response [173, 174]. IFNs have been the ideal cytokines to be applied clinically in a variety of human cancers [154, 155]. However, the results of IFN treatment of most solid tumors have been generally disappointing [175, 176]. The unsatisfactory performance of IFNs in cancer treatments has been in part attributed to the poor delivery of the protein to the tumor [160, 175]. Nevertheless, some reports demonstrated that combination of cytokine gene therapy with conventional chemotherapy or other types of cytokines show a favorable outcome compared to single cytokine gene therapy [155, 169]. In the future, combination gene therapy will likely become standard protocol for glioma therapy.

Viral-mediated delivery of single interleukins has not been as extensively investigated in GBM as in other cancers, but studies in this direction have definitely shown therapeutically relevant results [27]. In order to activate T-cells, a tumor cell must present tumor cell antigen in the context of MHC class I and simultaneously present the co-stimulatory antigen. Once the T-cell is activated, it up-regulates the functional high affinity IL-2 receptor and secretes IL-2 and −4. The T cell proliferates in response to autocrine signals from IL-2 and IL-4 resulting in an anti-tumor effect [9]. On the other hand, IL-12 is produced by phagocytes, B cells and DCs following an encounter with infectious agents. IL-12 acts on T and NK cells by enhancing the generation and activity of cytotoxic lymphocytes and inducing the proliferation and production of cytokines. IL-12 is the major cytokine responsible for the differentiation of helper T cells, which are potent inducers of IFN-γ. IFN-γ, in turn, has a strong effect on the ability of phagocytes and DCs to produce IL-12 [177, 178]. Consequently, IL-12 has a potent adjuvant activity in generating anti-cancer effects [177].

In a rat glioma model, recombinant vaccinia virus expressing the cytokines IL-2 or IL-12 resulted in tumor growth inhibition after intratumoral injection [179]. Combination gene therapies of IL-2 and IL-12 proved to be more effective in increasing the NK, Natural killer T, and Mac-1^+^ phagocytic cell populations in blood as well as increased IFN-γ, and TNF-α expression in tumors without significant toxicity. Non-replicating adenoviral-associated virus and replicating HSV have also been used to distribute IL-12 in experimental models of malignant gliomas [180–182] and the results showed a significant inhibition of tumor growth and a local immune reaction including increased IFN-γ expression, microglial activation, and recruitment of T and NK lymphocytes (Figure 3). Colombo *et al.* reported using combined delivery of IL-2 with HSV-TK for 12 patients with recurrent malignant glioma [183]. All patients received a stereotactic or open surgical intratumoral injection of retroviral vector-producing cells which express both the *HSV-TK* gene and *IL-2* gene following by intravenous GCV. The 12-month progression-free survival rate and overall survival rates were 14% and 25%, respectively. Although there was a marked increase of circulating IFN-γ, TNF-α, IL-2, and IL-10, only minor adverse events were noted [183].

IL-4 is produced by Th2-type T lymphocytes, mast cells, and basophils [184]. IL-4 increases surface MHC class II antigen expression in B cells and stimulates the growth of both helper and cytotoxic T cells, including tumor infiltrating lymphocytes [185–187]. Therefore, IL-4 is thought to have a potent anti-tumor effect *in vivo* against various types of cancer by inducing an immune response [188–191]. Okada *et al*. demonstrated a synergistic effect using a retrovirally transduced *IL-4* plus *HSV-TK* gene in a rat intracranial glioma model [192]. This resulted in a clinical trial using *IL-4/HSV-TK* gene-modified autologous glioma cells or fibroblasts [193]. Conditionally replicative oncolytic HSV carrying IL-12 gene therapy will start in Phase I clinical trial to determine the safety in patients with recurrent glioma (Table 1).

#### Tumor suppressor gene therapy

**Figure 4:**
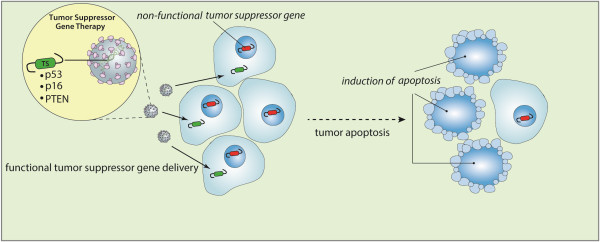
**Strategy of tumor suppressor gene therapy.** Tumor suppressor gene therapy aims to reprogram tumor cells by restoring the function of a tumor suppressor gene lost or functionally inactivated in cancer cells, subsequently inducing cell cycle arrest or apoptosis.

The aim of tumor suppressor gene therapy is to restore the function of a tumor suppressor gene lost or functionally inactivated in cancer cells (Figure 4). Commonly, they regulate diverse cellular activities including cell-cycle checkpoints, detection and repair of DNA damage, cell proliferation and apoptosis.

The tumor suppressor gene, *P53* is located on chromosome 17p and encodes a 393 amino acid protein [194–196]. As the name “Guardian of the Genome” suggests, p53 plays a critical role in causing cell cycle arrest and apoptosis in response to a variety of cellular stress such as radiation exposure [197–200]. P53 directly contributes to DNA repair, and inhibition of angiogenesis [195, 199]. The most well-characterized function of p53 is the inhibition of abnormal cell growth. Many factors contribute to the control of p53 activation and its downstream response which are crucial in the prevention of tumor development.

Inactivation of p53, which is one of the most commonly mutated tumor suppressor genes in glioma, plays a critical role in glioma progression [113, 201]. Alteration of p53 is seen in approximately 50% of grade II and III glioma, 25-30% of primary GBM, and 60-70% of secondary GBM [202]. Tumor suppressor gene therapy using p53 in glioma was first tested by delivering this gene using a replication-deficient AV [27]. The most commonly used non-replicating adenoviral vector for *p53* gene transfer is the type 5 AV in which the E1 region is replaced with the cDNA of the *p53* gene and is driven under the control of a CMV promoter (Ad5CMV-p53) [113, 203–209]. Removal of the E1 region makes the virus replication-defective, reducing the possibility of widespread, uncontrolled, systemic infection. The CMV promoter is particularly active in human cells and significantly increases gene expression [113]. Restoration of the functionally intact *TP53* gene induced robust apoptosis of the infected cells and inhibited cellular proliferation both *in vitro* and *in vivo* [113, 204, 207] *.* There was marked growth inhibition of implanted gliomas and significant prolongation of survival in preclinical models [203, 208–210]. *P53* gene therapy might also suppress angiogenesis of GBM [211]. Several studies have suggested that Ad5CMV-p53 may be most effective when combined with radiation and chemotherapy [205, 206, 212, 213]. An additional *in vitro* study suggested that combined p53 transfection with other type of genes such as *Fas Ligand*, *granulocyte–macrophage colony-stimulating factor* and *B7-1* enhances apoptosis and inhibits cell growth [214, 215].

Collectively, these preclinical results led to a Phase I clinical trial of Ad5CMV-p53 gene therapy in recurrent malignant glioma. Eligible patients underwent stereotactic injection of the virus pre- and post-resection through an implanted catheter. Immunohistochemical staining for p53 protein confirmed exogenous p53 expression within the nuclei of glioma cells and induced apoptosis in all specimens examined. One limitation was revealed when transduced cells were only found within a short distance from the injection site. In terms of outcome, the median progression free survival and overall survival were 13 and 43 weeks, respectively. Of note, one patient was alive more than three years after treatment without evidence of recurrence. The clinical toxicity was minimal without reaching maximum tolerable dose [216]. Limitations of p53 gene therapy are considered to be insufficient gene transfer, lack of bystander effect and tolerance arising from genetic heterogeneity of glioma [27, 217].

Another tumor suppressor gene therapy candidate is p16^INK4A^, which causes cell cycle arrest at the G1-S transition point by stabilizing the hypo-phosphorylated status of the Rb protein [200, 218]. The over-expression of p16 gene using a recombinant replication-deficient adenovirus significantly reduced glioma cells invasion as a result of decreased activity of matrix metalloprotease-2 [218].

The third tumor suppressor gene therapy candidate is Phosphatase and Tensin Homologue (PTEN) which contains a central catalytic phosphatase core domain that negatively regulates PI3K by dephosphorylating from phosphatidylinositol-triphosphate to phosphatidylinositol-diphosphate [200]. Inactivation of PTEN is seen in 40% to 50% of all gliomas resulting in aberrant activation of PI3K activity and downstream signaling pathways [219]. Expression of PTEN in GBM cells caused tumor cell apoptosis and decrease glioma cell proliferation *in vitro* [220, 221]. Furthermore, adenoviral PTEN expression demonstrated an anti-angiogenic response in preclinical study [221].

## Conclusion

GBM remains one of the most frequent and most clinically challenging primary brain tumors encountered by neurosurgeons. Over the past decades, current standard treatments have evolved to include maximal surgical resection followed by adjuvant radiation and chemotherapy. Unfortunately, these therapies have yet to cure patients with GBM. However, several remarkable advances in the treatment of GBM have occurred. Surgery augmented with powerful imaging techniques is aimed at maximal resection of tumor tissue without causing new neurological deficits. Effective reduction of tumor mass can allow the patients to achieve a better overall survival. Stereotactic radiosurgery or radiotherapy that attempt to enhance the effect of other treatment modalities has been developed. In addition to current standard conventional chemotherapy for GBM, advancements in our understanding of the pathogenesis of GBM and the molecular aberrations in GBM has led to new era of exciting possibilities for the treatments of GBM.

In the last few decades, a considerable amount of research utilizing gene therapy for glioma has been published in *in vitro* and in animal models. Although most of these methods have demonstrated success in *in vitro* and in pre-clinical studies, a majority of patients in early clinical studies have ultimately failed to demonstrate significant survival for the treatment of GBM. This is partly because experimental therapies in early clinical study are usually applied to patients with recurrent or advanced GBM disease, who have often been treated with multiple treatment regimes, including radiation or chemotherapy (TMZ) or both. These patient tumors are likely already beyond a curable level and a modest response can be expected even with a well established therapy at the time of the trial and this likely contributes to the low success rate observed in clinical trials. For proper evaluation of efficacy, patients with earlier stages of GBM need to be enrolled in clinical studies.

A number of specific difficulties are associated with gene therapy for GBM including: limited transduction efficiency of the viral vectors; lack of a delivery system that bypasses the BBB; inability to distinguish tumor cells from normal cells; and the selective expression of a transgene in a therapeutically controlled manner (Table 2). The histological heterogeneity of the cell population within the tumor is considered to be another major impediment. The restricted intratumoral distribution of the viral vector still remains an issue for obtaining optimal clinical efficacy due to the infiltrative nature of GBM. Greater vector stability, as well as prolonged therapeutic transgene expression, might result in more efficacious treatment of GBM. Therefore, in order to improve gene therapy efficiency, new vectors should overcome the limited infiltration and transgene expression that occurs after administration. In addition to viral vectors, stem cells have been successfully used to deliver therapeutic gene products to primary and secondary invasive brain tumors. Theyhave demonstrated their usefulness in combined gene therapy such as suicide gene therapy and cytokine gene therapy. Another important property is the stem cell’s ability to migrate toward infiltrative GBM tumors even when administered peripherally. Stem cells may become an important vector option in gene therapy for GBM.

**Table 2:** **Comparison of gene therapy strategies for GBM**

Suicide gene therapy	Oncolytic gene therapy	Cytokine mediated gene therapy	Tumor suppressor gene therapy
● Synergistic therapeutic efficiency of conventional treatment	● Additional therapeutic transgenes available	● Local augmentation of the immune response inside the brain	● Anti-angiogenesis effect
● Bystander effect	● Selective toxicity	● Combination therapy with other types of gene therapy available	● Synergistic therapeutic efficiency of conventional or other of gene therapy
● Selective cytotoxicity	● Higher transduction efficiency	● Reduce tumor vascularization and invasion	
▲ Transduced cells may become resistant to the prodrug	▲ Suppression of virus by host immune response	▲ Lack of antigen presenting cells inside the brain	▲ Resistance from the inherent genetic heterogeneity
▲ Low efficiency for distribution	▲ Cerebral inflammation and edema	▲ CNS toxicity	▲ Lack of bystander effect
▲ Low delivery to target cells		▲ Poor delivery of a gene to the tumor	▲ Poor gene transfer
▲ Limited prolonged efficacy			

●, Advantages; ▲, Disadvantages.

Gene therapy alone will not likely provide a cure of GBM, at least not in the near future. Since GBM is a heterogeneous tumor, therapeutic blocking of one or two pathways may simply result in the activation of alternative pathways leading to continuous tumor progression. Therefore, simply replacing a single lost gene (i.e. tumor suppressor gene) never brings about success in treatment of GBM. However, gene therapy was able to demonstrate significant anti-cancer effects in other types of cancers including melanoma, hepatocellular carcinoma, and squamous cell carcinoma in head and neck, suggesting that gene therapy still has a great potential for inclusion in GBM treatment protocols. Furthermore, multiple options are now available, including more complex systems involving combination of suicide genes or oncolytic virotherapy with immunological or tumor suppressor genes, selectively replicating viruses, and non-viral vectors. It is likely that viral gene therapy when administered with other treatment modalities, such as advanced radiation therapy and molecular targeted therapy, would demonstrate greater efficacy over the treatment with viral agents alone. For instance, brain radiotherapy can disrupt the BBB facilitating enhanced viral delivery. Patient safety does not appear to be a significant concern in the clinical gene therapy studies in patients with GBM. Although an ideal vector has yet to be developed, future treatment of GBM will likely incorporate multimodal therapy to study synergistic relationships between improved gene therapy and current radiation and chemotherapeutic regimens. A significant obstacle to the development of multidrug combination clinical trial protocols includes legal entanglements that preclude a drug from one company from being combined with a second. The design of combination protocols including gene therapies may ultimately alleviate some of these legal obstructions. As the field of gene therapy moves forward, the use of gene therapy in the treatment of GBM will become an increasingly promising area of research to support the therapeutic regimens of surgery, radiation, and chemotherapy.

## Abbreviations

5-FC: 5-fluorocytosine
5-FU: 5-fluorouracil
AAV: Adeno-associated virus
AV: Adenovirus
BBB: Blood brain barrier
bp: Base pair
CD: Cytosine deaminase
CEA: Carcinogenic embryonic antigen
CMV: Cytomegalovirus
CNS: Central nervous system
CSC: Cancer stem cell
DC: Dendritic cell
DNA: Deoxyribonucleic acid
E1B: Early region 1B
Edm: Edmonston strain
EIF-2α: Eukaryotic initiation factor-2 alpha
Flt3L: Fms-like tyrosine kinase-3 ligand
GBM: Glioblastoma multiforme
GCV: Ganciclovir
GSC: Glioma stem cell
HSV: Herpes simplex virus
ICP: Infected cell protein
IFN: Interferon
IL: Interleukin
IRES: Internal ribosome entry site
MHC: Majorhistocompatibility
MV: Measles virus
NDV: Newcastle disease virus
NK: Natural killer
PKR: Protein Kinase R
PTEN: Phosphatase and tensin homologue
Rb: Retinoblastoma
RR: Ribonucleotide reductase
TGF: Transforming growth factor
TK: Thymidine kinase
TMZ: Temozolomide
UL: Unique long
UPRT: Uracil phosphoribosyltransferase.
